# Peptic Ulcer and Gastric Cancer: Is It All in the Complex Host–Microbiome Interplay That Is Encoded in the Genomes of “Us” and “Them”?

**DOI:** 10.3389/fmicb.2022.835313

**Published:** 2022-04-25

**Authors:** Angitha N. Nath, R. J. Retnakumar, Ashik Francis, Prakash Chhetri, Namrata Thapa, Santanu Chattopadhyay

**Affiliations:** ^1^Rajiv Gandhi Centre for Biotechnology, Trivandrum, India; ^2^Manipal Academy of Higher Education, Manipal, India; ^3^Department of Zoology, Biotech Hub, Nar Bahadur Bhandari Degree College, Tadong, India

**Keywords:** *H. pylori*, genome, microbiome, peptic ulcer disease, gastric cancer, virulome, immunome

## Abstract

It is increasingly being recognized that severe gastroduodenal diseases such as peptic ulcer and gastric cancer are not just the outcomes of *Helicobacter pylori* infection in the stomach. Rather, both diseases develop and progress due to the perfect storms created by a combination of multiple factors such as the expression of different *H. pylori* virulence proteins, consequent human immune responses, and dysbiosis in gastrointestinal microbiomes. In this mini review, we have discussed how the genomes of *H. pylori* and other gastrointestinal microbes as well as the genomes of different human populations encode complex and variable virulome–immunome interplay, which influences gastroduodenal health. The heterogeneities that are encrypted in the genomes of different human populations and in the genomes of their respective resident microbes partly explain the inconsistencies in clinical outcomes among the *H. pylori*-infected people.

## Introduction

No pathogenic bacteria created a bigger spark among microbiologists, gastroenterologists, and science enthusiasts than *Helicobacter pylori* upon its discovery from the human stomach. Once the colonization of this bacterium in the harshly acidic human gastric milieu was confirmed by histology and culture by Robin Warren and Barry Marshall in 1983, within the next 20 years, the total number of publications on it surpassed the total number of publications on *Salmonella*, which was discovered in 1855 (Warren and Marshall, 1983; Harry et al., 2001). Once the role of *H. pylori* as the causative agent of gastritis, peptic ulcer disease (PUD), and gastric cancer (GC) was firmly established, the World Health Organization (WHO) classified it as Class I carcinogen (first among all bacteria) in 1994 (IARC, 1994); gastritis and PUD became curable with triple therapy (a proton pump inhibitor and two antibiotics); gastric mucosa-associated lymphoid tissue (MALT) lymphoma became the first malignancy to be reversed with antimicrobial agents (Malfertheiner et al., 2014); and both Warren and Marshall were awarded the Nobel Prize in Physiology and Medicine in 2005. However, at the same time, it was also realized that *H. pylori* colonization in the human stomach is remarkably common and is not just restricted to patients suffering from gastric and duodenal diseases. A study showed that in 2017, 4.4 billion people (57.9% of the global population at that time) were infected with *H. pylori* (Hooi et al., 2017). Although *H. pylori* remains colonized in the stomach of a major fraction of the population, only a small subset of people, typically 10–20% of the infected population, suffer from severe gastroduodenal diseases such as PUD and GC, but the reasons for the inconsistencies in clinical outcomes were not precisely understood (Bauer and Meyer, 2011). Data emerged in the past four decades suggest us to appreciate that PUD and GC may have multiple and complex etiologies such as *H. pylori* infection, polymorphisms in human cytokine genes, dysbiosis in the gastric and intestinal microbiome, the influence of geography, climate, and altitude, lifestyles such as diet, smoking, and alcohol consumption, and perturbations that are imposed by different medicines (Alexander et al., 2021). In this mini review, we discussed how complex and functional host–microbe interplays that determine the gastroduodenal health are encoded in the human genome and in the genomes of trillions of microbes (including *H. pylori*) that populate the human gastrointestinal tract.

## *Helicobacter pylori*: Our Friendly Foe Since Ancient Time

*H. pylori* is one of the oldest human pathogens known. A 5,300-year-old iceman mummy (named Ötzi), excavated from the Italian Alps, had *H. pylori* DNA in the stomach (Maixner et al., 2016). Interestingly, unlike most human pathogens, *H. pylori* exclusively colonizes humans (Mendall and Northfield, 1995). *H. pylori* colonization is acquired during the initial years of life by an intrafamilial manner through oral/fecal–oral route and remains colonized for decades before causing any severe diseases (Brown, 2000). Most (80–90%) of the infections, however, either remain asymptomatic and unnoticed or cause noticeable gastritis. Typically, the antral predominant gastritis is associated with *H. pylori*-induced gastrin secretion followed by increased H^+^ secretion by parietal cells and predisposes to PUD. However, in direct contrast, long-term *H. pylori* infection leads to hypochlorhydria due to decreased H^+^ secretion by the parietal cells and this allows the growth of several other bacteria (discussed later) (McColl et al., 1998). This cascade of events leads to atrophic gastritis, which eventually progresses to intestinal metaplasia, intraepithelial neoplasia, and GC (Barra et al., 2021).

Continuous changes, as part of evolution within the human stomach, have occurred and accumulated in different *H. pylori* genomes for thousands of years along with human migrations, which has started 60,000 years ago in different clades, followed by settlements in different geographical regions (Correa and Piazuelo, 2012). The modern *H. pylori* strains, which have successfully “coevolved” with humans, are classified into distinct populations—hpAfrica1 (subpopulations—hspWAfrica and hspSAfrica), hpAfrica2, hpEastAsia (subpopulations—hspAmerind, hspEAsia, and hspMaori), hpEurope, hpNEAfrica, hpAsia2, and hpSahul (Falush et al., 2003; Linz et al., 2007; Moodley et al., 2009). The above *H. pylori* populations exhibit distinct geographical predisposal. hpEurope is distributed in the Middle East, India, Iran, and Europe. hspWAfrica is present in Western Africa, while hspSAfrica and hpAfrica2 are present in South Africa. hpNEAfrica circulates in Nilo-Saharan speakers of northern Nigeria, Sudan, Ethiopia, and Somalia. hspEAsia is distributed among East Asians, while hspMaori is seen in Taiwan’s Aboriginal, Melanesian, and Polynesian populations. hspAmerind is present among Native Americans. hpAsia2 is seen in populations of Malaysia, Thailand, Bangladesh, and northern India. Strains from Papua New Guinea, Aboriginals, and Australia belong to the hpSahul population (Correa and Piazuelo, 2012).

Overall, *H. pylori* is one of the most genetically diverse species among bacterial pathogens (Suzuki et al., 2012). Its diversity is due to a higher level of spontaneous mutations occurring within the restricted gastric niche, a higher frequency of horizontal gene transfer, and natural competence (Israel et al., 2001). Impaired DNA repair, integration of acquired DNA into the “plasticity zones,” and higher intraspecific recombination also contributed to diverse genetic forms (Fernandez-Gonzalez and Backert, 2014). *H. pylori* remains a very successful human pathogen for centuries with a considerably lower proportion of terminal clinical outcomes and higher self-propagation across generations with a plethora of virulence factors that facilitate chronic colonization in the human stomach, where the pH is nearly 2. *H. pylori* virulence factors include urease, flagella, adhesins, and several effector proteins that lead to pathogenesis. One of the major effector proteins of *H. pylori* is the oncoprotein CagA, encoded by the *cagA* gene present in the 40 kb *cag* pathogenicity island (*cag*PAI). Inside the gastric cell, phosphorylated CagA interacts with Src homology-2 (SH2) domains of the host proteins such as CSK, Grb2, and SHP2, leading to altered cell proliferation and differentiation, cytoskeletal changes, and increased proinflammatory cytokines (IL-8) secretion *via* the NF-κB pathway (Papadakos et al., 2013; Hatakeyama, 2014). Another effector protein, the VacA, gets internalized by binding to the receptor protein tyrosine phosphatases (RPTPα and RPTPβ) and low-density lipoprotein receptor-related protein-1 (LRP1), resulting in cell vacuolation and cell deaths by apoptosis, necrosis, and autophagy (Foegeding et al., 2016; Chauhan et al., 2019).

**FIGURE 1:**
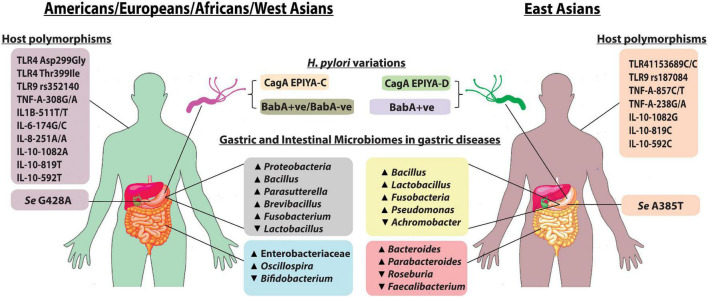
Pattern of gastric and intestinal microbiomes, host polymorphisms, and *H. pylori* type between East Asia and the rest of the world: the more virulent *H. pylori* CagA EPIYA-D is mostly found in East Asia when compared with *H. pylori* CagA EPIYA-C, which is predominant in the Western world (Yamaoka, 2008). Almost all strains from East Asia express BabA, while in West, BabA-positive strains as well as a minority of BabA-negative strains are present. FUT2, encoded by the *Se* gene, is required for the synthesis of LeB antigen (BabA-binding host molecule). Mutations in the *Se* genes, *Se* G428A and *Se* A385T, are found to inactivate and to reduce the activity of FUT2, respectively (Anstee, 2010). The alteration in gastric and intestinal microbiome composition is observed in *H. pylori* infection, and gastric diseases also exhibit regional variations as shown in the figure. Additionally, specific host immune gene polymorphisms in different populations predispose an individual susceptibility to *H. pylori* colonization and gastric diseases.

The capabilities of the *H. pylori* strains to establish colonization and to induce pathogenic alterations in the stomach are greatly determined by the allelic types of virulence genes, which vary with geography (Table 1). For *vacA*, the alleles observed are *s1* (with subtypes *s1a* and *s1b*) and *s2* for the *vacAs* region; *i1* (with subtypes *i1a* and *i1b*) and *i2* for the *vacAi* region; *m1* (with subtypes *m1a*, *m1b*, and *m1c*) and *m2* for the *vacAm* region; *c1* and *c2* for the *vacAc* region; and *d1* and *d2* for the *vacAd* region (Trang et al., 2016; Alexander et al., 2021). CagA protein also shows distinct variations between strains circulating in different populations. The Western *H. pylori* strains carry CagA EPIYA-C, while the EPIYA-D is typically expressed by East Asian strains (Figure 1; Higashi et al., 2002). The *vacAs1i1m1cagA* + (particularly of East Asian-type CagA) strains are associated with aggressive clinical outcomes as compared to *vacAs2m2cagA* − strains. The diversities in virulence encoded in the *H. pylori* genomes in different populations are the major determinants of different clinical manifestations in different populations (Table 1; Chang et al., 2018). For example, the prevalence of GC is highest in East Asian countries, but is remarkably low in African countries (Rawla and Barsouk, 2019).

**TABLE 1:** Variations in *H. pylori* genotype, microbiome, and host genes and association with gastric diseases.

Variations in *H. pylori* genotype			Variations in microbiome			Variations in host immune-associated genes		
Region	Dominant *H. pylori* types	Associated condition	Region	Dominant microbiome	Associated condition	Region/ethnicity	Host gene polymorphisms	Associated condition
India	*vacA s1a m2* (Saxena et al., 2011)	PUD and GC	**Intestinal Microbiome**			Asians	TNF-A-857C/T and TNF-A-238G/A (de Brito et al., 2018)	GC
			India	Increase in *Oscillospira* (Devi et al., 2021)	PUD and GC			
				Decrease in *Bifidobacterium* (Devi et al., 2021)				
			**Gastric Microbiome**					
			East Asia	*Bacillus* (Cavadas et al., 2020)	GC			
							IL-10-1082G, IL-10-819C, and IL-10-592C (Kim et al., 2013)	GC
							TT genotype of IL-10-819C/T (Xue et al., 2012)	Protection against GC
						India	IL-1B-511TT (Chakravorty et al., 2006)	PUD
						Saudi Arabia	TLR4-*rs4986790* (A > G), TLR4-*rs4986791* (C > T), TLR10-*rs10004195* (A > T) (Eed et al., 2020)	*H. pylori* infection
			South Korea	High *Lactobacillus*, *Fusobacterium, Bacillus*, *and Pseudomonas* (Cavadas et al., 2020)	GC			
							TLR9-*rs352140* (C > T) (Eed et al., 2020)	*H. pylori* gastritis
Iran	*vacAs1,vacA m1, vacAs1m2* (Keikha et al., 2020)	PUD		Low *Achromobacter* (Cavadas et al., 2020)		Chinese	TLR4-rs11536889and TLR9-rs187084 (T > C) (Castaño-Rodríguez et al., 2013)	GC
							TLR4-*rs1927911, rs10759931*, and *rs10116253* (Castaño-Rodríguez et al., 2013)	Protection against GC
							TLR10-*rs10004195* (Castaño-Rodríguez et al., 2013)	Protection from *H. pylori* infection
East Asia	*vacA s1c m1 i1* *cagA* + (EPIYA-D) (Sahara et al., 2012)	PUD and GC	Vietnam	Higher *Achromobacter, Bacillus, and Pseudomonas* (Cavadas et al., 2020)	GC		CD14 260C/T polymorphism (Castaño-Rodríguez et al., 2013)	GC
							TLR10-*rs10004195* T allele (Castaño-Rodríguez et al., 2013)	*H. pylori* infection
				Lower *Lactobacillus*, *Fusobacterium* (Cavadas et al., 2020)				
			China	Increase in *Slackia exigua, Streptococcus anginosus, Peptostreptococcus stomatis, Dialister pneumosintes*, and *Parvimonas micra* (Coker et al., 2018)	GC	Japan	TLR4-*rs11536889* C allele with miR146A (Hishida et al., 2011)	Gastric atrophy
						Korea	NOD1 G796A (Kim et al., 2013)	*H. pylori*-induced gastric mucosal inflammation
						Turkey	NOD1 796 A/A (Castaño-Rodríguez et al., 2013)	Gastric atrophy Antral intestinal metaplasia
			Malaysia	*Streptococcus* (Khosravi et al., 2014)	PUD			
						Kazak	IL-1B-511T/T allele, IL-1B-31C/C (Kulmambetova et al., 2014)	Gastritis
Latin America	*vacA s1m1* (Sugimoto and Yamaoka, 2009)	GC and PUD	**Intestinal Microbiome**			Caucasian	TLR4 SNP Asp299Gly and SNP Thr399Ile (Cheng et al., 2007)	GC
			Finland	High Enterobacteriaceae (Sarhadi et al., 2021)	GC			
Africa	*vacA s1m1* (Sugimoto and Yamaoka, 2009)	GC		Low *Bifidobacterium* (Sarhadi et al., 2021)				
			**Gastric Microbiome**					
			Europe	High *Bacillus* (Cavadas et al., 2020)	GC			
							TNF-A-308 G/A (de Brito et al., 2018)	
							IL-10-1082A, IL-10-819T, and IL-10-592T (Kim et al., 2014)	
						European	TLR10-*rs10004195* T allele (Mayerle et al., 2013)	Protection from *H. pylori* infection
							IL-1B-511T/-31T/IL-1RN*2 (El-Omar et al., 2000)	GC
			**Gastric Microbiome**					
			United States	High *Fusobacterium* and low *Lactobacillus* (Cavadas et al., 2020)	GC	Brazil	IL-6–174G/C polymorphism (Gatti et al., 2007)	GC
							IL-8-251 A/A (Ramis et al., 2017)	PUD
						Mexican	TT genotype of IL-10-819C/T (Martínez-Campos et al., 2019)	Protection against GC
							IL-10-592C/A (Martínez-Campos et al., 2019)	Lower risk of GC

*The dominant H. pylori genotypes, the predominant members of the gastrointestinal microbiome, and host immune response-associated gene polymorphisms distributed in different geographical locations, along with corresponding disease association, are given in the table. The distribution of various vacA alleles in different regions of Europe and America shows the predominance of vacA s1b in Spain and Portugal, vacA s1a in Northern and Eastern Europe, and vacA s1a and vacA s1b in France, Italy, and North America (Van Doorn et al., 1999). The composition of gastric disease-associated gastrointestinal microbiome also varies significantly with geography. It is to be noted that several host gene polymorphisms confer protection against H. pylori infection as well as gastric diseases (GC—gastric cancer, PUD—peptic ulcer disease).*

## The Rewards and Penalties From the Microbial World Inside Us

Every living human body carries its unique microbiome composed of a few trillion microbes along with their respective genomes that express different proteins for carrying out metabolic functions (Cho and Blaser, 2012; Blaser, 2014; Gilbert et al., 2018). Every anatomical niche of a healthy individual has a distinct microbiome that helps in various physiological processes (Kennedy and Chang, 2020). Conversely, enrichment of a few bacterial taxa, which leads to dysbiosis in the microbiome, is deleterious to human health (Turnbaugh et al., 2007; Hullar et al., 2014). The microbiome composition of each niche for each individual varies due to several factors such as antibiotic usage, geography, diet, lifestyle, and *H. pylori* infection (Alexander et al., 2021).

Chronic colonization of the *H. pylori* in the stomach alters the local immune response, leading to dysbiosis in the gastric microbiome (Brawner et al., 2014). Proteobacteria and Firmicutes are predominant in the gastric microbiome of *H. pylori*-positive individuals, while Actinobacteria, Firmicutes, and Bacteroidetes were dominant in *H. pylori*-negative individuals (Andersson et al., 2008). Significant variations in the relative abundances of genera such as *Veillonella, Granulicatella, Neisseria, Fusobacterium, Prevotella, Actinomyces*, and *Streptococcus* were also observed between *H. pylori*-positive and *H. pylori*-negative individuals (Klymiuk et al., 2017). The gastric microbiome composition of patients with *H. pylori*-associated gastritis was almost exclusively dominated by *H. pylori*, whereas a high microbial diversity was observed in *H. pylori*-negative gastritis individuals and in normal individuals (Parsons et al., 2017; Gantuya et al., 2019). Notably, in a study from Malaysia, *Streptococcus* was isolated at a significantly higher frequency in PUD cases (Khosravi et al., 2014). On the other hand, in the gastric microbiome of patients with GC, the abundance of Helicobacteraceae was lower than that in patients with chronic gastritis (Eun et al., 2014). For patients with advanced stages of GC, a distinct lower abundance of *H. pylori*, but higher abundances of *Prevotella, Streptococcus, Veillonella*, and *Lactobacillus*, in the gastric microbiome was observed (Dicksved et al., 2009). An increased abundance of the family Lachnospiraceae and *Lactobacillus coleohominis* along with a decreased count of *Neisseria, Porphyromonas*, and *Streptococcus sinensis* showed an association with GC (Aviles-Jimenez et al., 2014). On the other hand, GC patients from China had enrichment of species such as *Slackia exigua, Streptococcus anginosus, Peptostreptococcus stomatis, Dialister pneumosintes*, and *Parvimonas micra* in the stomach (Coker et al., 2018). Furthermore, an overgrowth of nitrate-reducing bacteria in the atrophic stomach is attributed to the development of gastric cancer *via* the accumulation of N-nitroso compounds (Barra et al., 2021).

Even though *H. pylori* colonization is restricted to the stomach, an alteration in the normal intestinal microbiome is observed during *H. pylori* infection in the murine model and in human (Kienesberger et al., 2016; Dash et al., 2019). Although the mechanism remains poorly studied, it is possibly due to the *H. pylori*-induced hypochlorhydria and altered gastrointestinal immunity. *H. pylori* infection is associated with increased diversity in the intestinal microbiome (Yang et al., 2019; Devi et al., 2021). Children with *H. pylori*-associated gastritis showed a higher abundance of *Parabacteroides* and *Bacteroides*, and a lower abundance of *Faecalibacterium* and *Roseburia*, than the healthy control group (Benavides-Ward et al., 2018). In India, individuals with *H. pylori*-associated diseases showed a higher *Oscillospira* abundance in the intestinal microbiome, while their *Bifidobacterium* abundance was remarkably low (Devi et al., 2021). A lower *Bifidobacterium* abundance in the intestine was also observed among Finnish patients with GC along with a higher abundance of Enterobacteriaceae (Table 1; Sarhadi et al., 2021). The use of probiotics such as *Bifidobacterium* and *Lactobacillus* has been shown to moderately improve *H. pylori* eradication and reduce the side effects of antibiotics (Zhang et al., 2015). It is known that probiotic strains of *Lactobacillus* and *Bifidobacterium* also impart a protective effect against *H. pylori* infection (Yang et al., 2021). *L. acidophilus* and *L. bulgaricus* decrease *H. pylori* adhesion to the gastric epithelial cells. Also, *L. bulgaricus* suppresses the secretion of proinflammatory cytokine IL-8 by gastric epithelial cells (Song et al., 2019).

Apart from *H. pylori* infection, gastrointestinal microbiome composition is affected by geographical variations and ethnicity, which indirectly influence the progression of gastric diseases (Figure 1; Gupta et al., 2017). Gastric microbiome analysis showed a higher abundance of Proteobacteria in Europeans, while a higher abundance of Firmicutes was observed in Asians. GC cohort from South Korea had a higher abundance of *Lactobacillus*, followed by *Fusobacterium*, and a lower abundance of *Achromobacter*, while Vietnamese cohorts had an opposite trend (Cavadas et al., 2020). *Bacillus* and *Pseudomonas* were found to be dominant in GC cohorts from both regions. Patients with GC from the United States and Europe had a relatively higher abundance of *Bacillus, Parasutterella, Brevibacillus*, and *Fusobacterium*. Patients with GC from the United States also had a lower abundance of *Lactobacillus* (Cavadas et al., 2020). Although the link between dysbiosis and gastroduodenal diseases is noticeable, the functional mechanisms involved in the process remained poorly described to date.

## Our Defenses and Predispositions Are Encrypted in Our Genomes

*H. pylori* is present in the human stomach since ancient times, but only a subset of the *H. pylori*-infected population is genetically predisposed to gastroduodenal diseases. Colonization of *H. pylori* in the human stomach is recognized by the body with pathogen recognition receptors (PRR) such as nucleotide-binding oligomerization domain (NOD) and Toll-like receptor (TLR) and eventually leads to the expression of cytokines, such as tumor necrosis factor (TNF) and interleukin 8 (IL-8) (Deforge and Remick, 1991; Amarante-Mendes et al., 2018). Genome-wide association study showed that polymorphisms in the PRR and cytokine genes among individuals from different geographical and ethnic backgrounds critically affect the immune response to *H. pylori* and clinical outcomes (Table 1 and Figure 1; Mommersteeg et al., 2018). A case-control study from Saudi Arabia showed that patients with TLR4-*rs4986790* (A > G), TLR4-*rs4986791* (C > T), and TLR10-*rs10004195* (A > T) have a significant association with *H. pylori* infection, and TLR9-*rs352140* (C > T) is connected with *H. pylori*-associated chronic gastritis (Eed et al., 2020). However, for people with Chinese ethnicity, the CC genotype of TLR4-*rs11536889* and TLR9-*rs187084* (T > C) is associated with an increased risk of GC, while TLR4-*rs1927911, rs10759931*, and *rs10116253* were found to confer protection against GC (Castaño-Rodríguez et al., 2013). Also, a Chinese population with TLR10-*rs10004195* polymorphism exhibited protection against *H. pylori* infection (Tang et al., 2015). A significant association has been identified between *H. pylori*-related GC and TLR4 SNPs, Asp299Gly, and Thr399Ile in a Caucasian population (Cheng et al., 2007). NOD1 796G > A polymorphism is linked to gastric mucosal inflammation in *H. pylori*-infected Korean population, while NOD1 796A/A genotype increases risk of gastric atrophy and antral intestinal metaplasia in a Turkish population (Kara et al., 2010; Kim et al., 2013). TNF-A-308G/A polymorphism increases the risk of GC in Caucasians, while TNF-A-857C/T and TNF-A-238G/A polymorphism increases the risk of gastric tumorigenesis in Asians (Yang et al., 2014; de Brito et al., 2018). Europeans with IL-1B-511T/-31T/IL-1RN*2 have a high risk of GC, while in a Kazakh population, IL-1B-511T/T and IL-1B-31C/C increase the risk of gastritis (El-Omar et al., 2000; Kulmambetova et al., 2014). However, in India, IL-1B-511TT genotype was higher in *H. pylori*-infected patients with PUD (Chakravorty et al., 2006). IL-6-174 G/G polymorphism in a Brazilian population is associated with a higher GC risk, while IL-8-251 A/A shows a higher risk of PUD (Gatti et al., 2007; Ramis et al., 2017). It was observed that IL-10-592T, IL-10-819T, and IL-10-1082A alleles increased the risk of GC in Caucasians, while IL-10-592C, IL-10-819C, and IL-10-1082G alleles were associated with GC risk in Asians (Kim et al., 2014). The TT genotype of IL-10-819C/T was shown to confer protection against GC in Mexican and Asian populations (Xue et al., 2012; Martínez-Campos et al., 2019).

## Virulome–Immunome: The Overlooked Interplay

Since the origin of anatomically modern humans in Africa and their subsequent migration, parallel evolutions and diversifications have also occurred to trillions of microbes (including *H. pylori*), which remained inhabited on and in the human body over the entire periods of human migrations and settlements. The pattern of genetic distance between different *H. pylori* strains from different populations reflects the migration pattern and its coevolution with its host (Falush et al., 2003; Domínguez-Bello et al., 2008). *H. pylori* remains attached to the human stomach with its adhesins. The blood group antigen-binding adhesin (BabA) on the bacterial surface binds to a difucosylated ABO/Lewis b (LeB) antigen present on the surface of human gastric epithelial cells (Huang et al., 2016). Both *H. pylori* BabA and human LeB are diverse proteins, which show remarkable variations with geography and ethnicity that subsequently affects colonization and clinical outcomes (Figure 1). Similarly, colonizing in the human gastrointestinal tract by members of the microbiome depends on the respective adhesin–receptor interactions, which are yet to be described. Also, while all virulence genes within the genomes of different *H. pylori* strains are well studied, the total virulence-associated proteins encoded in the genomes of all members of the gastric and intestinal microbiome, the virulome, which must have an effect on the gastric epithelium, are completely overlooked till date.

Like microbial virulome, human immunome, the total immune response genes present to protect against the invading pathogens, is also not sufficiently understood. It is known that the gastric niche contains several PRRs such as TLR and NLR along with antimicrobial peptides and mucins (Peek et al., 2010). The presence of antimicrobial peptides such as cathelicidins, hepcidins, and defensins and O-glycosylated protein mucin plays an important role in protecting the gastric epithelium from bacterial colonization (McGuckin et al., 2011; Li and Yu, 2020). A recent study also demonstrated the importance of galectin-3 in gastric epithelium against *H. pylori* infection (Park et al., 2016).

The virulome–immunome interplay is inevitable and possibly contributes to determining the clinical outcomes in the context of *H. pylori* infection and microbiome alteration. Bacterial pathogens are capable of modulating the host immune responses and cause damage. For example, *Propionibacterium acnes* (associated with lymphocytic gastritis) is known to produce short-chain fatty acids (SCFAs) such as propionate and butyrate that induce NKG2D–NKG2DL (natural killer group 2 member D) and the proinflammatory cytokine IL-15 that promote the progression to GC (Montalban-Arques et al., 2016). Further studies are necessary to understand how the gastrointestinal virulome manipulates the human immunome in the context of PUD and GC.

## Conclusion

PUD and GC are complex diseases that develop with the influence of multiple factors. All major contributory factors–the *H. pylori* virulence, the gastrointestinal microbiome along with their virulome, and the microbe-responsive human immunome–show tremendous unevenness among different individuals and among different geographic regions, which is also linked to human migrations and settlements. The present inconsistencies that we observe in the clinical outcomes within the *H. pylori*-infected population settled in different locations have their roots in the combined evolutions of human immunome along with *H. pylori* virulence and gastrointestinal virulome, which is being continued for at least 60,000 years.

Human gastrointestinal health is undeniably the consequence of dynamic interplay between the gastrointestinal virulome and the host immunome. Recent studies suggest that a shift in this equilibrium has far-reaching effects on the progression of gastroduodenal diseases. Engineering the gastrointestinal microbiome by interventions like probiotics to modulate the host immune response may turn out to be an efficient strategy for the management of a spectrum of gastroduodenal diseases in the future, particularly in this era of growing antimicrobial resistance. However, further multidisciplinary approaches are required for uncovering the complex mechanisms so that more specific and effective microbiome-based therapies can be designed in the future.

## Author Contributions

SC conceived the idea. AN, RR, AF, and PC contributed to writing the manuscript. NT and SC edited the manuscript. All authors contributed to the article and approved the submitted version.

## Conflict of Interest

The authors declare that the research was conducted in the absence of any commercial or financial relationships that could be construed as a potential conflict of interest.

## Publisher’s Note

All claims expressed in this article are solely those of the authors and do not necessarily represent those of their affiliated organizations, or those of the publisher, the editors and the reviewers. Any product that may be evaluated in this article, or claim that may be made by its manufacturer, is not guaranteed or endorsed by the publisher.
